# Immunohistochemical estimation of cell cycle phase in laryngeal neoplasia

**DOI:** 10.1038/sj.bjc.6603262

**Published:** 2006-07-11

**Authors:** P Chatrath, I S Scott, L S Morris, R J Davies, K Bird, S L Vowler, N Coleman

**Affiliations:** 1 MRC Cancer Cell Unit, Hutchison/MRC Research Centre, Cambridge CB2 2XZ, UK; 2 Royal National Throat Nose & Ear Hospital, 330 Grays Inn Road, London WC1X 8DA, UK; 3 Department of Surgery, Addenbrooke's Hospital, Cambridge CB2 2QQ, UK; 4 Centre for Applied Medical Statistics, Department of Public Health and Primary Care, University Forvie Site, Robinson Way, Cambridge CB2 2SR, UK

**Keywords:** cell cycle regulation, laryngeal squamous cell carcinoma, minichromosome maintenance proteins, S-phase fraction

## Abstract

We previously developed an immunohistochemical method for estimating cell cycle state and phase in tissue samples, including biopsies that are too small for flow cytometry. We have used our technique to examine whether primary abnormalities of the cell cycle exist in laryngeal neoplasia. Antibodies against the markers of cell cycle entry, minichromosome maintenance protein-2 (Mcm-2) and Ki67, and putative markers of cell cycle phase, cyclin D1 (G1-phase), cyclin A (S-phase), cyclin B1 (G2-phase) and phosphohistone H3 (Mitosis) were applied to paraffin-embedded sections of normal larynx (*n*=8), laryngeal dysplasia (*n*=10) and laryngeal squamous cell carcinoma (*n*=10). Cells expressing each marker were determined as a percentage of total cells, termed the labelling index (LI), and as a percentage of Mcm-2-positive cells, termed the labelling fraction (LF). The frequency of coexpression of each putative phase marker was investigated by confocal microscopy. There was a correlation between Mcm-2 and Ki67 LIs (*ρ*=0.93) but Mcm-2 LIs were consistently higher. All cells expressing a phase marker coexpressed Mcm-2, whereas Ki67 was not expressed in a proportion of these cells. The putative phase markers showed little coexpression. Labelling index values increased on progression from normal larynx through laryngeal dysplasia to squamous cell carcinoma for Mcm-2 (*P*=0.001), Ki67 (*P*=0.0002), cyclin D1 (*P*=0.015), cyclin A (*P*=0.0001) and cyclin B1 (*P*=0.0004). There was no evidence of an increase in the LF for any phase marker. Immunohistochemistry can be used to estimate cell cycle state and phase in laryngeal biopsies. Our data argues against primary cell cycle phase abnormalities in laryngeal neoplasia.

Squamous cell carcinoma of the larynx is the most common neoplasm of the head and neck (Watkinson *et al*, 2000). Its progression follows a series of steps through increasing grades of dysplasia to malignancy (Shanmugaratnam, 1991). Despite an understanding of this sequential process, little clarity exists as to the precise histopathological distinction between different grades of dysplasia. Moreover, algorithms for the management of laryngeal malignancy do not adequately take into account the biological aggressiveness of a lesion, which can result in small early tumours recurring many times despite an apparently comprehensive treatment protocol (Gale *et al*, 2000). Features predictive of tumour aggression and poor ultimate survival are not consistently demonstrated on histopathological evaluation (Gale *et al*, 2000), giving rise to an inevitable degree of subjectivity and variability in diagnosis (Hellquist *et al*, 1999). Given the potentially mutilating and functionally disabling nature of aggressive laryngeal surgical interventions, a drive towards earlier detection and more reliable stratification of laryngeal dysplastic and malignant lesions would be welcome and indeed is essential if further attempts to improve quality of life and outcome are to be realised (Chatrath *et al*, 2003).

Estimation of cell cycle phase can be used to predict clinical behaviour and response to chemotherapy in a variety of malignancies (Huuhtanen *et al*, 1999; Sandquist *et al*, 2000; Pelletier *et al*, 2001). Flow cytometry is a widely described technique to analyse cell cycle phase in tumour tissue (Baretton *et al*, 1990; Hixon *et al*, 1995; Nakamura *et al*, 1995; Pinto *et al*, 1997; Cascinu *et al*, 1998; Villanacci *et al*, 1998), but this approach has not entered routine clinical practice. Reasons for this include a lack of standardised procedures, difficulties in data interpretation, tissue heterogeneity and complexity of the equipment required for analysis. We aim to develop a simple immunohistochemical method to allow cell cycle phase studies to be performed on routine paraffin-embedded laryngeal tissue, using methods that we have already demonstrated to be effective in colorectal tissue (Scott *et al*, 2003).

To achieve this, we have utilised a panel of antibodies against proteins involved in cell cycle regulation. These include minichromosome maintenance protein 2 (Mcm-2) and Ki67, markers of cell cycle entry and therefore cell cycle ‘state’ in tissues (Maiorano *et al*, 1996; Newlon, 1997; Kearsey and Labib, 1998; Musahl *et al*, 1998; Tanaka and Diffley, 2002). Minichromosome maintenance protein 2 is one of six MCM proteins (MCMs 2–7) that assemble in the prereplication complex and are essential for DNA replication in eukaryotic cells (Kearsey and Labib, 1998). All six proteins are abundant throughout the cell cycle but are broken down rapidly on differentiation and more slowly in quiescence (Musahl *et al*, 1998). Antibodies against Mcm-2 have previously been shown to detect more cycling cells in laryngeal tissues than other ‘proliferation’ markers such as Ki67 (Chatrath *et al*, 2003), and immunohistochemical staining for Mcm-2 and/or Mcm-5 has been shown to be of value in identifying malignant or premalignant lesions in a range of clinical specimens (Williams *et al*, 1998; Freeman *et al*, 1999; Davies *et al*, 2002; Going *et al*, 2002; Chatrath *et al*, 2003; Scott *et al*, 2003; Sirieix *et al*, 2003; Scott *et al*, 2004).

Other proteins detected in our method include putative markers of cell cycle phase, levels of which peak during specific stages of the cell cycle, followed by rapid degeneration as the cell progresses to the next phase (Hunter and Pines, 1991; Hunter and Pines, 1994; Musahl *et al*, 1998). These include cyclin D1, which is maximally expressed in mid-to-late G1-phase and, together with cyclin E, is involved in the G1 to S transition (Pines and Hunter, 1989). Cyclin A is expressed in S-phase with variable expression in G2-phase (Pines and Hunter, 1989; Sherr, 1996). Cyclin B1 is expressed as a cytoplasmic molecule in G2-phase but becomes nuclear in early M, until breakdown in early G1-phase (Nasmyth, 1996; Sherr, 1996). Histone H3 is phosphorylated maximally in mitosis and phosphohistone H3 is not detected following entry into G1-phase (Shibata and Ajiro, 1993). Antibodies against these markers might therefore enable *in situ* labelling of cells at all phases of the cell cycle, with the exception of early G1 for which no useful marker is available (Scott *et al*, 2003). Indeed, we have shown previously that in colorectal mucosa, only small numbers of epithelial cells coexpress putative markers of adjacent cell cycle phases and that the cell cycle phase distribution determined by our immunohistochemical approach compares well with phase analyses obtained by flow cytometry (Scott *et al*, 2003).

Here, we demonstrate that our approach can be applied to laryngeal dysplasia and SCC. Our data suggests that there are no primary cell cycle phase abnormalities in these conditions. Selected markers, particularly those of cell cycle entry, may ultimately prove of value in improving the clinical management of laryngeal neoplasia.

## MATERIALS AND METHODS

### Clinical specimens

Archival blocks of paraffin embedded, formalin-fixed human laryngeal tissue, obtained from surgical resection specimens, were retrieved in accordance with Local Research Ethics Committee guidelines.

The tissues examined represented normal laryngeal squamous epithelium taken from resection margins distant from tumour (*n*=8), various grades of laryngeal dysplasia (*n*=10) and laryngeal squamous cell carcinoma (SCC) (*n*=10). The dysplastic lesions were classified according to the Ljubljana classification (Hellquist *et al*, 1999): seven represented atypical hyperplasia (low-grade) and three carcinoma *in situ* (high-grade). None of the patients with SCC had received preoperative neoadjuvant therapy. Of the SCCs, four were well differentiated and six were moderately differentiated.

### Primary antibodies

We used mouse monoclonal antibodies against Mcm-2 (Mukherjee *et al*, 2001, dilution 1 : 10); Ki67, (DAKO, Ely, UK Mib-1 clone, dilution 1 : 10); Cyclin A, (Novocastra, Newcastle, UK, clone NCL Cyclin A, dilution 1 : 20); Cyclin D1, (Novocastra, Newcastle, UK, clone NCL Cyclin D1, dilution 1 : 50) and Cyclin B1, (DAKO, Ely, UK, clone V152, dilution 1 : 400), and a rabbit polyclonal against phosphohistone H3 (Upstate Biotechnology, Lake Placid, NY, USA, ref 06-570, dilution 1 : 300).

### Immunohistochemical staining of paraffin-embedded tissues

Sections (5 *μ*m) were cut onto aminopropyltriethoxysilane (APES) coated slides and processed for immunohistochemisty as described previously (Freeman *et al*, 1999). Antigen retrieval was achieved by pressure-cooking for 3 min in citrate buffer (pH 6.0), except for the cyclin D1 preparations, which required heating in a programmable microwave (MicroMED T/T Mega) for 30 min at 98°C.

Primary antibody (100 *μ*l) was applied in a humidified chamber at 4°C overnight with gentle shaking in 1% BSA/TBS with 0.1% Triton X-100. The slides were then washed in TBS containing 0.025% Triton X-100 and incubated for 1 h with biotinylated goat anti-mouse or goat anti-rabbit secondary antibody (DAKO, Ely, UK). A streptavidin-horseradish peroxidase system (DAKO, Ely, UK) with the substrate diaminobenzidine was used to develop the stain. The slides were then lightly counterstained with Harris' haematoxylin, dehydrated in increasing concentrations of alcohol and cleared in xylene. Coverslips were applied with DEPEX mounting medium (Gurr, BDH, Poole, Dorset, UK).

Negative controls were performed by omitting the primary antibody or using isotype-matched negative control antibodies (DAKO, Ely, UK). Sections of cervix showing various grades of intraepithelial neoplasia were used as positive controls (Freeman *et al*, 1999).

### Double labelling studies

Double labelling experiments were performed as described previously (Scott *et al*, 2003). For these studies we selected blocks of SCC (*n*=6), in which there were adjacent areas showing dysplasia and normal laryngeal epithelium. Serial sections were used to validate the results from each antibody combination. Briefly, in the first series of reactions, antibody against Mcm-2 or Ki67 was combined with each of four putative phase markers, cyclin D1, cyclin A, cyclin B1 and phosphohistone H3. We tested the hypothesis that if Mcm-2 identifies all cells in cycle, none of the cyclin or phosphohistone H3 antibodies should detect cells negative for Mcm-2. Similar considerations would apply to Ki67 as a potential marker of cycling cells.

The second series of reactions was designed to investigate the frequency of coexpression of the putative markers of cell cycle phase. Markers were paired as follows: phosphohistone H3-cyclin D1 (putative markers of mitosis and G1-phase); cyclin D1-cyclin A (G1-phase and S-phase); cyclin A-cyclin B1 (S-phase and G2-phase); cyclin B1-phosphohistone H3 (G2-phase and mitosis).

Where the primary antibodies had been raised in different species they were added together and incubated overnight. Following washing, both secondary antibodies (Alexa Fluro goat anti-mouse 488 and Alexa Fluro goat anti-rabbit 546; Molecular Probes) were added together and incubated for one hour. After further washing, slides were counterstained using 4,6-diamidino-2-phenylindole (DAPI) (Sigma), washed and mounted in fluorescent mounting medium (DAKO).

When the primary antibodies were both mouse monoclonals a different procedure was performed. Initially, one of the primary antibodies was applied alone. After washing, the sections were incubated with Alexa Fluro goat anti-mouse 488 (Molecular Probes), followed by a blocking step with F(ab)_2_ goat anti-mouse IgG fragments (Jackson Immuno Research Laboratories). A further washing step was then performed before incubation with the second primary antibody. Following a final washing step and incubation in Alexa Fluro goat anti-mouse 633 (Molecular Probes), the slides were counterstained and mounted as described above.

Images were viewed and assessed using a Zeiss Axioplan 2 confocal microscope at wavelengths of 488, 546 and 633 nm.

### Quantification of antibody staining

A quantitative indication of staining was determined for each marker by calculating the percentage of total epithelial cells that were immunopositive, to produce a labelling index (LI). In each case a minimum of 500 cells was evaluated. Staining intensity was not included in the assessment, as only relatively minor variations in staining intensity were observed. The LI for each cell phase marker was also expressed as a percentage of the number of Mcm-2-positive cells, to produce a labelling fraction (LF). Labelling fractions were determined to estimate the proportions of cells in cycle that were in each cell-cycle phase. Approximations of the percentage of cycling cells in S-phase, G2-phase and M-phase were derived from the cyclin A LF, the cyclin B1 LF and the phosphohistone H3 LF respectively.

Counts were repeated independently by three individual observers (PC, ISS, RJD) and in all cases an inter-observer variation of less than 5% was observed.

### Statistical analysis

Differences between Mcm-2 and Ki67 LIs were compared using the Bland and Altman limits of agreement analysis (Bland and Altman, 1999). Spearman's rank correlation coefficient (*ρ*) and its 95% confidence interval was used for correlation. Differences in the LIs and LFs for each marker on progressing from normal through dysplasia to SCC were assessed using the Jonckheere-Terpstra (J–T) test (Hodges and Lehmann, 1963). Pairwise comparisons from these data were made by comparison of the median values. All analyses were carried out in SPSS V11.0 (SPSS inc, Chicago, Illinois) and graphs plotted in SPSS and S-Plus 2000 (Mathsoft, Seattle, WA).

## RESULTS

### Expression of Mcm-2 and Ki67

In the normal larynx, Mcm-2 expression was confined to the basal layers of the epithelium, with the surface epithelium failing to show any Mcm-2 expression, as described previously (Chatrath *et al*, 2003). In lesions showing atypical hyperplasia (low-grade), Mcm-2 expression was confined to the lower third. In contrast, in lesions showing carcinoma *in situ* (high-grade), Mcm-2 was expressed throughout the epithelium. The distribution of Ki67 was the same as that of Mcm-2 (Chatrath *et al*, 2003) and there was a positive correlation between Mcm-2 and Ki67 LIs (*ρ*=0.93 (0.84, 0.97)). However, Bland and Altman limits of agreement analysis of the data for all three sample groups showed that Mcm-2 LIs were consistently greater than Ki67 LIs. The mean difference was 17.49, with limits of agreement of 1.59 (95% CI: −3.29, 6.97) and 33.40 (95% CI: 28.02, 38.77). The SCCs showed a median Mcm-2 LI of 82% (range 64–91) and a median Ki67 LI of 61% (range 42–89) (Chatrath *et al*, 2003). In keeping with our findings at other anatomical sites (Freeman *et al*, 1999), the highest LI values were observed in the less well differentiated SCCs.

### Expression of putative phase markers

#### Cyclin D1

**Figure 1:**
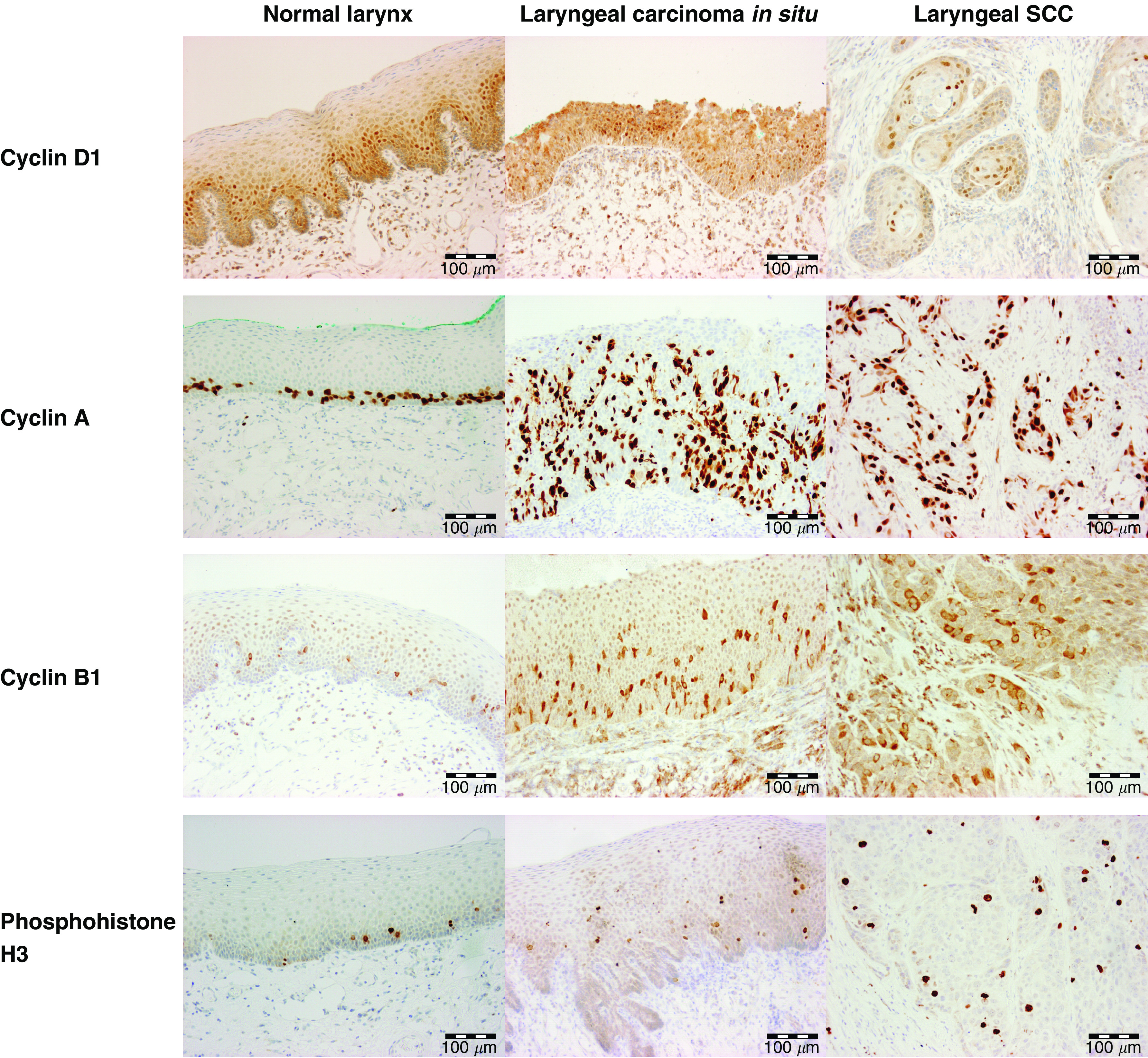
Distribution of putative cell cycle phase markers in laryngeal epithelium. Immunohistochemical staining showing the distribution (from top to bottom) of cyclins D1, A and B1 and phosphohistone H3 in normal larynx (left column), laryngeal carcinoma *in situ* (middle column) and laryngeal squamous cell carcinoma (right column).

The cyclin D1 LI increased on progression from normal (median=8.8%). through dysplasia (all cases; 11.4%) to SCC (33.3%) (*P*=0.015; J–T test). In all lesions, the distribution was similar to that of Mcm-2 (Figure 1).

#### Cyclin A

The tissue distribution of cyclin A was also similar to that of Mcm-2. In atypical hyperplasia, cyclin A was expressed in the basal and middle thirds of the epithelium, with full thickness expression in carcinoma *in situ* (Figure 1). In the SCCs, staining was seen throughout, but appeared to be greatest at the infiltrative tumour edges. The cyclin A LI showed a significant increase on progression from normal laryngeal epithelium (11.3%), through dysplasia (25.1%) to malignancy (27.5%) (*P*=0.0001; J–T test).

#### Cyclin B1

The staining pattern of cyclin B1 was either cytoplasmic, consistent with the identification of cells in G2-phase, or diffuse, consistent with M-phase. Labelling fractions were determined for cytoplasmic B1 staining only: these increased on progression from normal larynx (3.3%), through increasing grades of dysplasia (6.2%) to SCC (13.3%) (*P*=0.0004; J–T test). In the normal larynx, cyclin B1 expression was largely confined to the basal third of the epithelium (Figure 1) but with increasing grade of dysplasia, expression expanded to involve the full thickness of the epithelium (Figure 1). In SCC, staining was widespread (Figure 1).

#### Phosphohistone H3

Phophohistone H3 was detected in mitotic figures and in occasional nuclei presumed to be in prophase (Figure 1). No evidence of an increase in phosphohistone H3 LIs was detected between normal larynx (0.4%), dysplastic larynx (3.3%) through to squamous cell carcinoma (1.3%) (*P*=0.29; J–T test).

### Analysis of the coexpression of cell cycle markers by confocal microscopy

**Figure 2:**
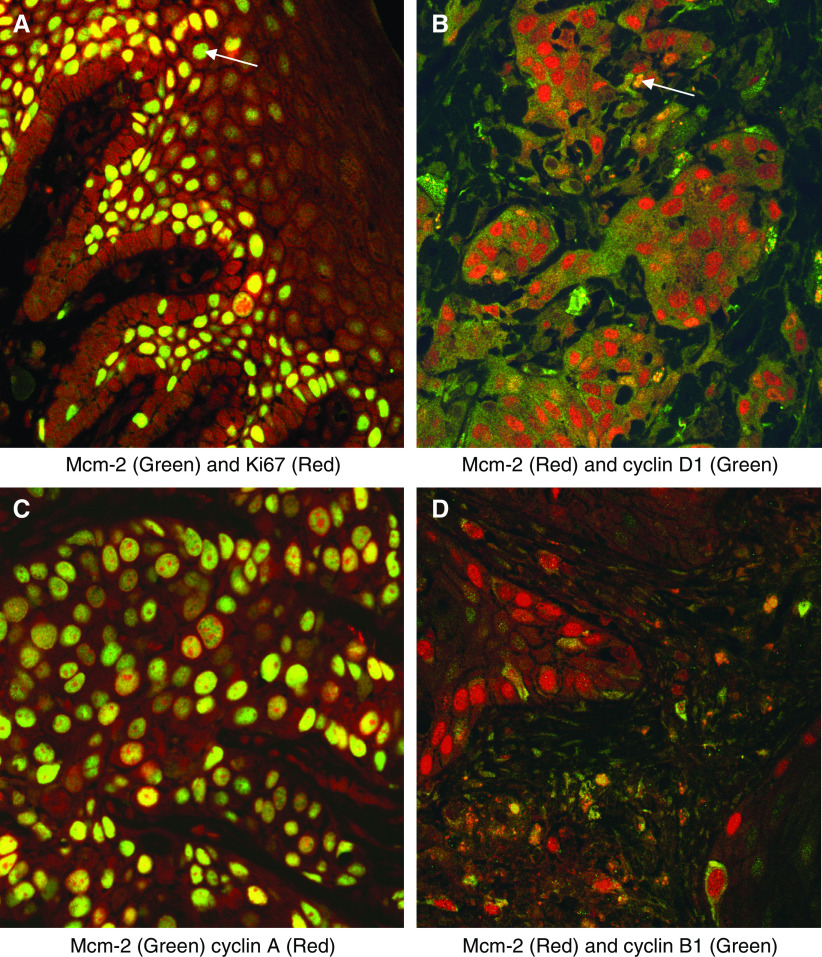
Double labelling fluorescent confocal microscopy analysis of markers coexpressed with Mcm-2 or Ki67. (**A**) Mcm-2 (green) and Ki67 (red) in normal laryngeal epithelium. Many cells coexpressed Mcm-2 and Ki67 (yellow), but occasional cells showed Mcm-2 expression in the absence of Ki67 (arrow). No cells showed Ki67 expression in the absence of Mcm-2. (**B**) Mcm-2 (red) detected all cells in a SCC coexpressing cyclin D1 (green), these cells appearing yellow (arrow). (**C**) Mcm-2 (green) was expressed in all cells expressing cyclin A (red) in a SCC. The cells expressing Mcm-2 in the absence of cyclin A appeared green. (**D**) In a SCC, nuclear Mcm-2 (red) was seen in cells showing cytoplasmic staining for cyclin B1 (green).

Double fluorescence labelling was first used to compare Mcm-2 expression with that of Ki67 (Figure 2A). While many cells coexpressed Mcm-2 (green) and Ki67 (red) giving a yellow signal, other cells showed Mcm-2 expression in the absence of Ki67. In view of the detailed counting performed on the numerous sections stained by immunohistochemistry (see above and below), formal quantification of staining in the confocal images was not performed. Nevertheless, in both SCC and normal laryngeal epithelium approximately 70–80% of Mcm-2-positive cells coexpressed Ki67, with the remainder being Ki67-negative. No cells were identified that expressed Ki67 in the absence of Mcm-2. In addition, in all six cases examined, Mcm-2 was detected in all cells expressing one of the putative cell cycle phase markers cyclin D1 (Figure 2B), cyclin A (Figure 2C), cyclin B1 (Figure 2D) and phosphohistone H3 (not shown). The percentages of Mcm-2-positive cells expressing each marker were within the range of the LFs calculated in the numerous sections stained by immunohistochemistry (see below).

**Figure 3:**
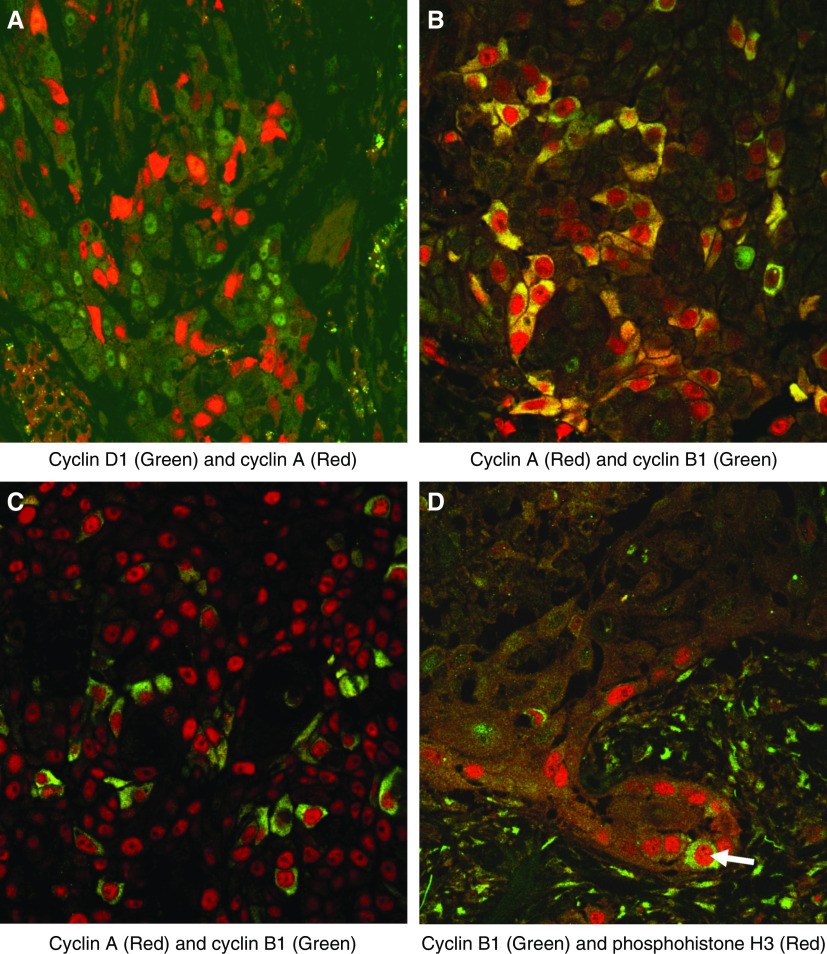
Double labelling fluorescent confocal microscopy analysis of coexpression of putative phase-specific markers. (**A**) There was negligible coexpression of cyclin D1 and cyclin A. (**B**) In dysplasia there was focal coexpression of cytoplasmic cyclin B1 (green) and cyclin A (red), the latter showing a nuclear and cytoplasmic distribution. An area of maximal coexpression is illustrated here. (**C**) In SCC, cytoplasmic cyclin B1 (green) was coexpressed by relatively few cyclin A positive cells (red). (**D**) Rare cells in SCC (arrow) showed nuclear expression of phosphohistone H3 (red) plus cytoplasmic staining for cyclin B1 (green), in keeping with cells at the G2/M transition.

We also performed double labelling to assess the degree of coexpression of the putative phase markers (Figure 3). There was virtually no coexpression of cyclin D1 and cyclin A in any sample (Figure 3A), the occasional cell showing coexpression presumably being at the G1/S transition. In normal larynx and dysplastic lesions there was coexpression of cyclin A and cytoplasmic cyclin B1 in approximately 50% of cyclin A-positive cells (approximately 5–10% of cells overall), suggesting that immunodetectable expression of cyclin A may extend further into G2 in laryngeal epithelium than we have observed previously in the colon (Scott *et al*, 2003) (Figure 3B). Many of the cells showing coexpression demonstrated both a nuclear and cytoplasmic distribution of cyclin A. In SCCs, however, coexpression of cyclin A and cytoplasmic B1 was much less prominent, amounting to only 5–10% of cyclin A positive cells (2–3% of cells overall) (Figure 3C). There was no colocalisation of nuclear cyclin B1 and cyclin A, suggesting that cyclin A expression is not detected in cells entering mitosis (Figure 3C).

There was minimal coexpression of cyclin B1 and phosphohistone H3 (<5% of phosphohistone H3-positive cells), the double-positive staining most likely representing cells in prophase or early metaphase (Figure 3D).

### Quantification of cycling cells

**Figure 4:**
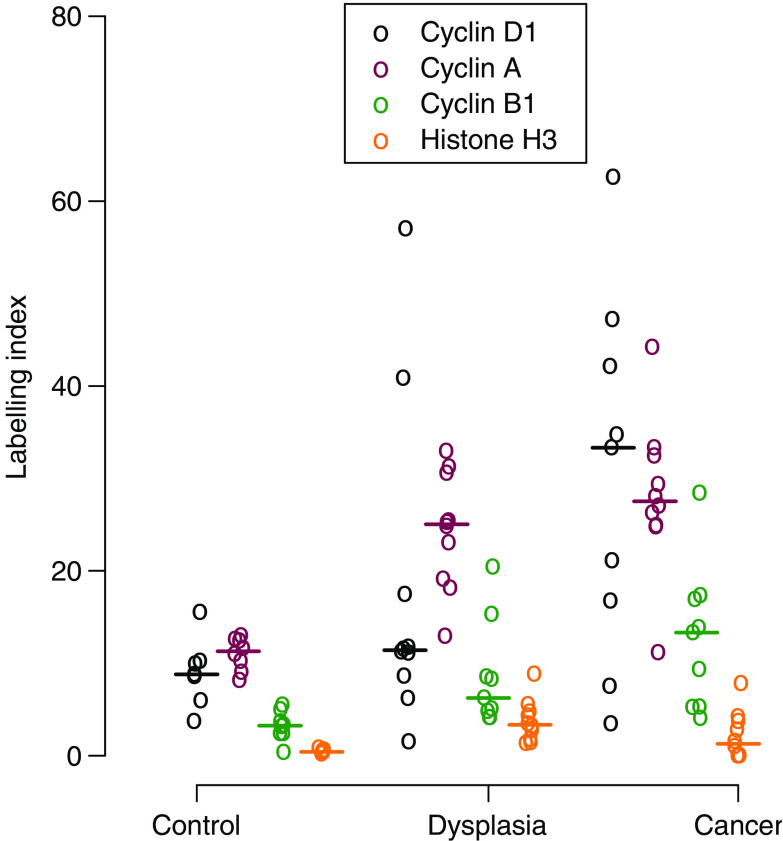
Labelling indices for putative cell cycle phase markers. Dot plot of cell cycle phase marker LIs in normal larynx (control), laryngeal dysplasia (dysplasia), and laryngeal squamous cell carcinoma (cancer). The LIs increased on progression from normal larynx through dysplasia to squamous cell carcinoma for cyclin D1 (*P*=0.015; J–T test), cyclin A (*P*=0.0001; J–T test), and cyclin B1 (*P*=0.0004; J–T test). There was no evidence of an increase in the LI for phosphohistone H3 (*P*=0.29; J–T test). Circles=individual values. Bar=median value.

The LI values increased on progression from normal larynx through laryngeal dysplasia to SCC for Mcm-2 (*P*=0.001), Ki67 (*P*=0.0002), cyclin D1 (*P*=0.015), cyclin A (*P*=0.0001), and cyclin B1 (*P*=0.0004) (Figure 4).

**Figure 5:**
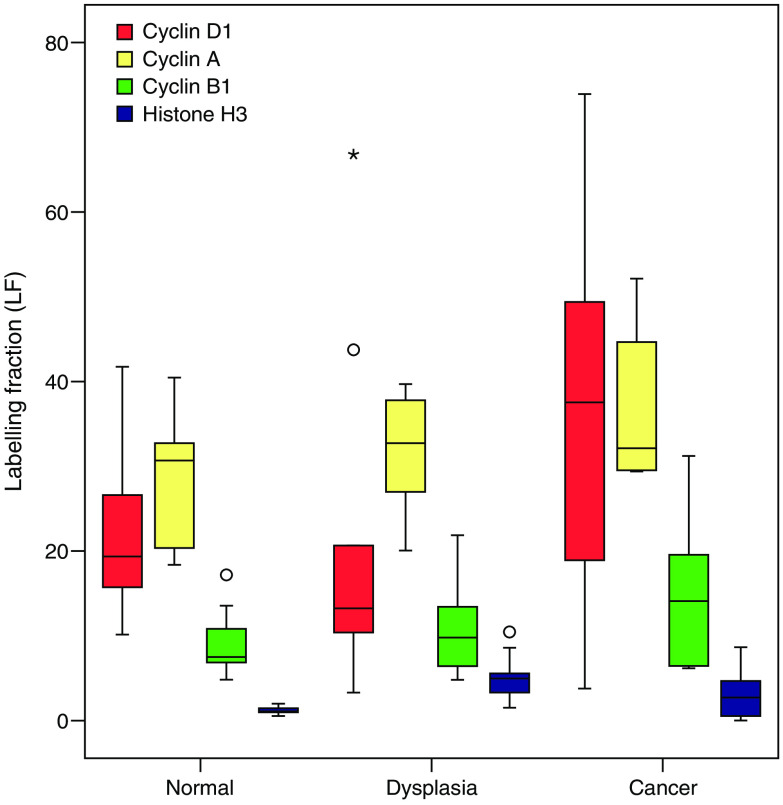
Labelling fractions for putative cell cycle phase markers in normal laryngeal epithelium (normal), laryngeal dysplasia and laryngeal squamous cell carcinoma (cancer). The frequencies of expression of putative cell cycle phase markers are depicted as a percentage of the number of Mcm-2-positive cells, to produce a LF. There was no evidence of an increase in LFs for any of the markers shown.

The frequency of expression of each putative phase marker was determined as a percentage of the number of Mcm-2-positive cells to produce a LF (Figure 5). There was no evidence of a difference in the LFs for cyclin D1 (*P*=0.33), cyclin A (*P*=0.15), cyclin B1 (*P*=0.11) or phosphohistone H3 (*P*=0.48) on progressing from normal larynx, through dysplasia to malignancy (J–T test).

## DISCUSSION

An accurate description of cell cycle parameters may be important in predicting the rate of growth, prognosis and response to chemoradiotherapy in laryngeal SCC, thereby complementing existing treatment protocols (Wilson *et al*, 2002). To achieve this by means of an immunohistochemical technique would represent a substantial advance, given the difficulties of flow cytometry in routine diagnostic practice. Flow cytometry is impractical for large scale analysis of laryngeal lesions, as biopsies are frequently only a few millimetres in size. Operator dependency and difficulties in standardisation between centres are also recognised problems. Nuclear integrity from paraffin sections is variable and the presence of inflammatory cellular infiltrates and blood vessels distort the analysis by the addition of large numbers of cells with a normal DNA complement. Aneuploid malignant cells may also result in an overestimation of the fraction of cells in G2/M (Monasebian and Ruskin, 1994).

Despite these technical limitations, there is some evidence that estimation of S-phase fraction by flow cytometry can predict the biological aggressiveness of tumours of the pharynx and larynx (Mohr *et al*, 1992; Monasebian and Ruskin, 1994; Stern *et al*, 1995; Wong *et al*, 1996; Myers *et al*, 1999). Hence, establishing a tool for estimating S-phase fraction in tissue sections may be of potential utility in improving diagnostic and treatment protocols for head and neck tumours in general, and possibly in monitoring the effectiveness of therapy. While alternative methods for measuring expression of cell cycle markers exist, including analysis of messenger RNA levels by microarray or quantitative PCR techniology, demonstration of protein levels by immunohistochemistry is likely to be most informative and most acceptable to general diagnostic laboratories.

In the present study, we did not compare our immunohistochemical data with that obtained by flow cytometry on the same samples. However, our overall technique has previously been validated in surgical resection specimens of colorectal carcinoma and ovarian neoplasms, in which we showed that our immunohistochemical model resulted in comparable cell cycle phase analyses to those obtained by flow cytometry (Scott *et al*, 2003; Scott *et al*, 2004). Nevertheless, confirmation of these observations by parallel flow cytometric and immunohistochemical analysis of laryngeal tumours will be important before commencing appropriately powered prognostic/predictive studies of larger numbers of samples.

Our present results show that Mcm-2 is expressed in a greater proportion of normal and neoplastic laryngeal epithelial cells than Ki67, consistent with previous data (Chatrath *et al*, 2003). Our findings suggest that whereas Ki67 can be expressed by cells in all phases of the cell cycle, it is not necessarily expressed by all cells in each phase and may therefore be less useful as a marker of cell cycle state than MCMs. Detailed analysis by double-labelling confocal microscopy suggests that the putative cell cycle phase-specific markers investigated in this study do not show substantial coexpression in laryngeal tissue, unlike findings in some tumour lines (Pines and Hunter, 1989; Nasmyth, 1996). The apparent phase-restricted expression of cyclin D1 suggests that previous observations of its expression throughout the cell cycle in tumour cell lines may not be reflected *in vivo* (Sherr, 1996). Alternatively, the limitations of immunohistochemistry in terms of sensitivity may restrict detection of cyclin D1 *in situ* to cells in mid-to-late G1, when its expression is maximal (Nasmyth, 1996). It is noteworthy that several antibodies recognising cyclin D1 are available and these may stain different proportions of cells in immunohistochemical preparations.

Cyclin A expression demonstrated minimal overlap with the expression of cyclin D1, as a putative marker of G1-phase. The degree of coexpression of cyclin A and cyclin B1 (a putative marker of G2-phase) was greater than we observed in our previous study of colorectal samples (Scott *et al*, 2003), although such coexpression was less prominent in laryngeal SCC than in dysplastic and normal laryngeal tissue. Previous analysis of colorectal cancer showed that cyclin A was not detected in any cell that was not actively replicating DNA (Scott *et al*, 2003), leading us to suggest that immunohistochemically detectable cyclin A expression could be used as a surrogate marker of S-phase in paraffin-embedded tissue.

Progression from normal laryngeal epithelium through dysplasia to SCC was associated with an increase in the LIs for each putative phase-specific marker other than phosphohistone H3, while the LFs remained consistent. These findings indicate that there is no evidence for any phase-specific cell cycle abnormality during neoplastic progression in laryngeal squamous epithelium. The particular elevation of cyclin A LI that we observed in laryngeal dysplasia is of uncertain significance, as no increase in cyclin A LF was seen in the same samples. Reassessment of this observation in a different, larger sample set is now required.

The immunohistochemical method used in this study offers numerous practical benefits. Unlike flow cytometry, examination of multiple sites from the available pathological specimen is possible, thereby allowing for a more representative evaluation of the inevitable heterogeneity that exists in laryngeal neoplasms. In particular, it is possible to identify and analyse the high-grade, poorly differentiated areas that are likely to exert the greatest influence on outcome. While our method may not be applicable to the very smallest samples, our experience to date is that an adequate number of sections can be obtained from laryngeal biopsies. Should larger scale studies confirm the present data, it will be of great interest to investigate the value of MCMs and other cell cycle markers in predicting prognosis and response to chemoradiotherapy regimes in laryngeal dysplasia and malignancy. The ease and reproducibility of the technique that we describe would enable such work to be performed in most diagnostic histopathology laboratories.
